# Anti-hepatitis B core antigen testing with detection and characterization of occult hepatitis B virus by an in-house nucleic acid testing among blood donors in Behrampur, Ganjam, Orissa in southeastern India: implications for transfusion

**DOI:** 10.1186/1743-422X-7-204

**Published:** 2010-08-27

**Authors:** Rajesh Panigrahi, Avik Biswas, Sibnarayan Datta, Arup Banerjee, Partha K Chandra, Pradip K Mahapatra, Bharat Patnaik, Sekhar Chakrabarti, Runu Chakravarty

**Affiliations:** 1 Defence Research Laboratory, Tezpur, Assam-784001, India; 2 Division of Infectious Diseases and Immunology, Saint Louis University, St Louis, 63104 MO, USA; 3 Department of Pathology & Laboratory Medicine, Tulane University School of Medicine, New Orleans, 70112 LA, USA; 4 ICMR Virus Unit, Kolkata, ID & BG Hospital Campus, Kolkata, India; 5 National Institute of Cholera and Enteric Diseases, Kolkata, India; 6 Red Cross Blood Bank, M.K.C.G. Medical College, Behrampur, Orissa, India; 7 Department of Chemistry, Jadavpur University, Kolkata, India

## Abstract

**Background:**

Occult hepatitis B virus (HBV) infection might transmit viremic units into the public blood supply if only hepatitis B surface antigen (HBsAg) testing is used for donor screening. Our aim was to evaluate the prevalence of occult HBV infection among the HBsAg negative/antiHBc positive donations from a highly HIV prevalent region of India.

**Methods:**

A total of 729 HBsAg negative donor units were included in this study. Surface gene and precore region were amplified by in house nucleic acid test (NAT) for detection of occult HBV infection and surface gene was analyzed after direct sequencing.

**Results:**

A total of 220 (30.1%) HBsAg negative donors were antiHBc positive, of them 66 (30%) were HBV DNA positive by NAT. HBV DNA positivity among 164 antiHBc only group, was 27.1% and among 40 antiHBs positive group was 30.0%. HBV/D (93.3%) was predominant and prevalence of both HBV/C and HBV/A was 3.3%. Single or multiple amino acids substitutions were found in 95% samples.

**Conclusion:**

Thus, a considerable number of HBV infected donors remain undiagnosed, if only HBsAg is used for screening. Addition of antiHBc testing for donor screening, although will lead to rejection of a large number of donor units, will definitely eliminate HBV infected donations and help in reducing HBV transmission with its potential consequences, especially among the immunocompromised population. The HBV genetic diversity found in this donor population are in accordance with other parts of India.

## Background

Detection of hepatitis B surface antigen (HBsAg) in blood is diagnostic for hepatitis B virus (HBV) infection. In the blood bank, screening for HBsAg is carried out routinely to detect HBV infection. Notably, donations from donors in seroconversion stage, from chronic HBV carriers with low circulating HBsAg level as well as from donors infected with some mutant HBV, may not be detected by the currently employed HBsAg assays. Thus among the common blood borne virus infections the risk of transfusion transmitted infection is highest for HBV [1]. Use of sensitive molecular assays has led to the detection of potentially infectious HBV DNA in the liver, serum, or both, in individuals without detectable HBsAg in circulation and has been termed "occult HBV infection" (OBI). It has often been explained by low levels of HBV DNA or mutations in the 'a' determinant region, (which contains a dominant B-cell epitope for anti HBs response), or undetectable HBsAg in circulation [2]. High frequencies of HBV DNA positivity have been described among antiHBc only sera, when antiHBc is the only seromarkers for HBV infection, in absence of antiHBs or HBsAg [3].

India has an estimated, more than 40 million chronic carriers [4]. Despite testing for HBsAg in blood donors, transfusion-associated HBV continues to be a major problem in India, especially among multiply transfused patients [5]. In India, detection of HBV infection among blood donors is carried out by HBsAg screening by commercial enzyme immuno assay. However, this strategy has sometimes failed due to the presence of occult HBV infection.

According to National AIDS control organization (NACO) reports (2008) Ganjam district in Orissa, southeastern India is amongst one of the highest HIV prevalent districts of India. Although the route of transmission of HIV and HBV are the same, data on HBV prevalence from southeastern Indian region is rare. Only one study on HBV infection was reported from Orissa, which deals with the prevalence of HBsAg among the blood donors and reported 1.13% HBsAg positivity [6]. Occurrence of OBI varies in different parts of India (from 0% in Chandigarh to 21% in Kolkata) [5,7-9]; but it has not been assessed from highly HIV prevalent Ganjam district. Furthermore, occult HBV infection has been linked at least in part to the genetic diversity of the viral Strains [10]; but data on HBV variability in blood donors from this region is not yet available. Understanding HBV evolution and strain diversity in the blood donor population could help to devise a better screening strategy for blood donors and help in developing more efficient ELISA method [11].

HBV has been classified into nine serotypes, ayw *(ayw1, ayw2, ayw3 *and *ayw4*), ayr, adw (*adw2 *and *adw4*), adr, and eight genotypes from A to H on the basis of complete genome and partial S gene sequence analysis [12-15]. The genotype distribution pattern in India is heterogeneous; HBV/D is the most predominant genotype across the country. In northern India, HBV/A and HBV/D are reportedly present in almost equal proportion [16], even though a recent study [17] showed a predominance of HBV/D similar to that documented from southern [18] and western [19] parts of India. Interestingly, in eastern India, although HBV/D is predominant & HBV/A as well as HBV/C is also reported in considerable proportion among blood donors [20,21].

In this study, we aimed to determine the prevalence of anti-HBC positivity in blood donations from Berhampur. Furthermore we also wanted to estimate the frequency of HBV-DNA in antiHBc-positive donations and distributation of HBV genotype/subgenotype along with mutations in S-gene region, to assess whether supplemental antiHBc testing may bring additional safety to blood products.

## Materials and methods

### Study subjects

The Red Cross blood bank in the city of Behrampur is the only blood bank in Ganjam district. It collects blood units from voluntary blood donation camps covering the most parts of the district. From December 2008 to July 2009, 729 HBsAg negative samples were collected from the Red Cross blood bank, Berhampur, Ganjam in Orissa for this study. The samples were from both urban and rural areas.

### Serological analysis

HBsAg (Biomerieux, Boxtel, The Netherlands), antiHBs and antiHBc (Hepanostika anti-HBc Uni-Form, Biomerieux, Boxtel, The Netherlands) were tested by commercial ELISA. All the AntiHBc positive samples were tested repeatedly and only those which have given repeated positive result were considered as antiHBc positive. Samples positive for either anti-HCV (Ortho-Clinical Diagnostics, NJ, USA) and/or antiHIV (Biomerieux, Boxtel, The Netherlands) were excluded from the study. Sera from patients were stored at -80°C, and are thawed once for serological examinations. The study was approved by Institutional ethical committee.

### Serum HBV DNA isolation, detection, sequencing, quantification

An in house nucleic acid tests (NAT) to directly detect the presence of HBV DNA using a combination of amplification and detection techniques similar to HCV RNA was employed [22]. HBV DNA isolation and amplification by in house NAT were done from surface region as described earlier [21,23]. Guidelines for avoiding false positive results [24] were followed strictly. Samples repeatedly positive for HBV DNA by surface gene amplification, were directly sequenced using Big Dye Terminator mix and a model ABI PRISM 3100xl sequencer (Applied Biosystems, Foster City, USA) as described previously [21]. Viral load was measured by using the real time Taqman PCR assay (Applied Biosystems SDS 7000, Foster City, USA) with lower detection limit 10^2 ^copies [25].

### Sequence analysis

The amino acids (aa 64-160) of the immunodominant region of the 60 HBsAg (-) isolates were compared with the reference sequences of each genotype. Genotypes/subgenotypes were ascertained as described by Norder et al. (2004) [13] and Banerjee et al. (2006) [20].

## Results

### Prevalence of occult infection

The demographic, serological and virological characteristics are shown in Table 1. A total of 729 (691 male, 38 female, mean age 33.3 ± 9.9) tested HBsAg negative were included in the study. The majority of the blood donors were males. Among them 220 (30.1%) were found to be antiHBc positive, of whom 66 (30%) were HBV DNA positive by nested PCR for surface region. Among 220 antiHBc positive donors 40 (18.2%) were antiHBs positive and 164 (74.5%) were anti-HBs negative. Among antiHBs positive donors, who are supposedly protected against HBV infection, 12/40 (30.0%) were HBV DNA positive. Furthermore, a significantly higher percentage of HBV DNA positivity (46.6%) among younger age group (age = 18-25) donors was observed (data not shown). When these samples were further subjected to PCR amplification using primers for precore region, only 23/65 (35.3%) samples were found to be positive. Among the 66 HBV DNA positive samples, viral load could be estimated in 40 samples because of insufficient samples. Most of the samples in which HBV viral load could be estimated, have <10^4 ^copies/ml.

**Table 1:** Overall demographic, serological, and virological characteristics of AntiHBs (+)/antiHBc (+) group among the blood donors

Features		Total
HBsAg (-ve)		729

Age years (mean ± S.D)		33.3 ± 9.9

Male/female		691/38

AntiHBc (%)		220/729 (30.1%)

PCR Positive (%)		66/220 (30.0%)

Surface region only (%)		42 (63.6%)

Precore/Surface gene		24 (36.3%)

**HBV genotype by sequencing (%)**		

A		2/60 (3.3)

C		2/60 (3.3)

D		56/60 (93.3)

**Features**	**HBsAg(-)/antiHBc(+)(n = 220)**	

	AntiHBs(+) (n = 40)*	**AntiHBc only (n = 164)**

**HBV DNA positive (%)**	12	45

Age in years (Mean ± S.D.)	29.8 ± 8.17	31.0 ± 13.4

Gender		
Male	11	44
Female	1	1

Surface gene sequencing	11/12	41/45

Viral load (n)	8	32
<10^4 ^copies/ml	7	31
≥10^4 ^copies/ml	1	1

Genotype/Subgenotype		
A/A1	1	1
C/C1	1	1
D/D1	1	1
D/D2	3	6
D/D3	5	32

*AntiHBs status of 16 samples were not detected due to unavailability of serum

### Genotype/Subgenotype Distributation

**Figure 1:**
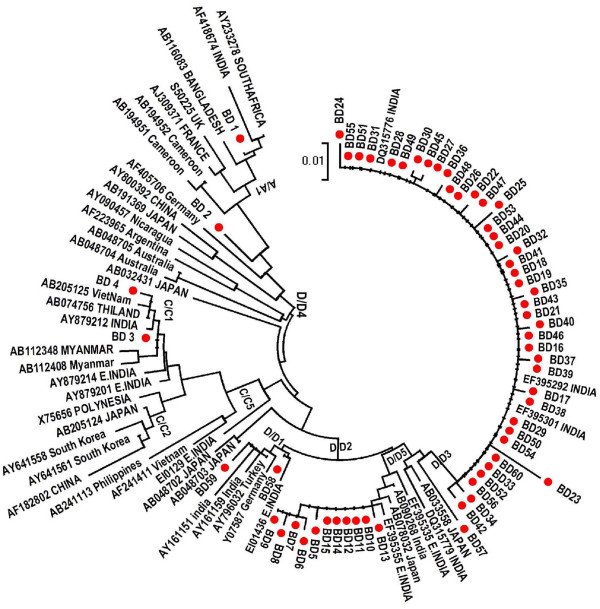
**The phylogenetic tree of HBV based on the partial S gene sequences of 60 (BD) isolates, from South Eastern India (marked with solid shapes)**. A phylogenetic tree constructed using NJ method, based on partial S gene sequences retrieved from the DDBJ/EMBL/GenBank. The number represents the percentages of bootstrap replicates (of 1,000 totals) for the node. The capital letters A to H designate HBV genotypes and the subgenotypes for each genotype were also included during the construction of the tree. Reference sequences are denoted by their accession number and the country from which the strain were isolated.

A total of 60 amplicons from the 66 HBV DNA positive samples could be sequenced and analyzed for genotyping. Phylogenetic analysis showed three genotypes (HBV/A, HBV/C, HBV/D) circulating in the study population (fig 1). HBV/D (94.3%) was predominant, in addition HBV/C (3.3%) and HBV/A (3.3%) (Table 1) was also detected. HBV/D was highly divergent and distributed mainly in three sub genotypes, HBV/D1, HBV/D2 and HBV/D3. Subgenotype D3 was most frequent among these occult HBV cases (n = 43). On the other hand, only one subgenotype of genotype A (HBV/A1 (Aa)) and one subgenotype C (HBV/C1 (Cs)) was found. HBV/A, subtype adw2 and HBV/D, subtype ayw2 and ayw3 from the present study clustered with the genotype A and D sequences previously reported from India. Similar to Eastern India, genotype C isolates with adr subtype from southeastern India clustered with HBV/Cs subgroup found in South East Asian countries rather than subgenotype Ce found in the Far East like China, Japan, and Korea.

### Amino acids substitutions in surface gene

Variability of surface gene in 60 HBsAg (-ve)/AntiHBc (+ve) individuals were studied (aa 64-163). Among HBsAg (-) samples, single or multiple amino acids substitutions were found in 57 of 60 samples (95.0%). The most common amino acid substitution found within MHL region was T125 M (42/45, 93.3%), mostly in HBV/D3. Other substitutions at codon G71 D, L95 S, M103I, P111L, S113A, S114P, T115I, T116P, S117G, T118R, A128V, T127A and were also found. Moreover, few novel substitutions, not reported previously, were detected among HBsAg (-) samples. Subtype ayw3 was more frequent than ayw2 among HBV/D samples. Subtype could not be ascertained in one genotype D sequence (BB67) due to substitution present at 127 amino acid position.

A stop codon found in one sample in HBV/D with a presence of single nucleotide substitution from thymidine (T) to adenosine (A) at 207th nucleotide (nucleotide position 362 from the unique *Eco RI *site) of the HBsAg ORF. Deduced amino acid sequence of the corresponding substitution showed a change from TGT (cysteine) to TGA (stop) at amino acid position 69 of HBsAg. Phylogenetic analysis of the S gene revealed that isolate is HBV/D, subgenotype D2 and subtype *ayw3*.

## Discussion

In India screening for HBsAg by ELISA is at present the only mandatory test for detection of HBV infection in blood donations. Several reports around the world showed detection of HBV DNA in blood of HBsAg-negative individuals and suggested transmission of HBV infection by blood transfusion from HBsAg negative occult HBV carriers [26,27]. Thus, evaluation of other HBV markers might reduce these risks.

Varying prevalence of antiHBc, a marker for exposure to HBV infection, has been reported from different parts of India; ranging between 8%-18% of total donor population. In the present study from Behrampur, Ganjam in Orissa, about 30.1% of total donations (220 of 729) was antiHBc positive indicating a very high rate of exposure to HBV infection among the blood donors from this region.

The Ganjam district in the state of Orissa in southeastern India records very high rate of migration in search of livelihood. Among the 3.1 million people living in 24 blocks in the district, people from 22 blocks depend on migration for their income [28]. Here, the men remain away from home for eight to nine months at a stretch, in their sexually most active period [28]. This also leads to a number of women left in Ganjam being involved in promiscuous habits [28]. This unusual matrix puts migrants and their families at risk of sexually transmitted infections at various points [28]. Notably around 50 per cent of the HIV positive people in Orissa are from Ganjam and most of them are migrant labourers and there is high prevalence of HIV among antenatal clinic attendees (3.25%), [28]. Thus, the high rate of antiHBc prevalence, indicating exposure to HBV-a sexually transmitted infection, among the blood donors of Ganjam district is not unusual.

Prevalence of occult HBV infection was also high among antiHBc positive donations from the present study, 30% percent of antiHBc positive donations (66/220) being HBV DNA positive. Furthermore, among the 220 antiHBc positive donations 40 were antiHBs positive and are supposedly protected against HBV infection. Notably, 12/40 (30.0%) of antiHBs positive donations were HBV DNA positive, even one of them was with viral load ≥10^4 ^copies/ml. Among the 164 antiHBc only donors, 45 (27%) were HBV DNA positive (Table 1). The higher proportion of OBI found among the younger group (18-24 & 25-34) bears special significance as they are in the sexually active group and might possibly be related to the problem of frequent migration for livelihood. However, this requires further study.

The frequency of occult HBV infection varied considerably from different parts of the world according to the prevalence of HBV in the population. Prevalence of OBI is high in high HBV prevalence zone of the world and low in low HBV prevalence zone of the world. Studies from other parts of India reported occult HBV infection ranging from 21% in Kolkata (Eastern India), 20.87% in New Delhi (Northern India) to 0% in Chandigarh (Northwestern India) [7-9]. A study from Japan [29] reported DNA positivity of 38% in 19 of 50 anti-HBc reactive samples. While that from North America found 3.7%, HBV DNA positive among 107 anti-HBc positive/anti-HBs negative samples [30]. Notably due to the low viral load in majority of these samples, detection of OBI required sensitive DNA amplification techniques.

There is increasing evidence that OBI is associated with chronic liver disease and HCC [31,32], in addition to being a source of transmission of HBV by blood transfusion or orthotropic liver transplantation [33]. Therefore, the high rate of OBI among blood donors of Ganjam district is of serious concern. As these donors have the potential to transmit HBV contaminated blood through the public blood supply even after donor screening for HBsAg. Thus, our data indicates OBI as an emerging infection hindering safety of blood transfusion in this community. Ideally, HBsAg negative individuals who are either antiHBc negative or those who are antiHBc positive/antiHBs positive/HBV DNA negative should be selected as regular blood donors to minimize transmission due to occult hepatitis B infection. This will help in the reduction of the transfusion associated transmission due to OBI; but will increase the testing costs.

Routine blood donor screening for antiHBc has been implemented in some countries resulting in a decrease in the risk of post-transfusion HBV infection [30]. Hence, in our resource poor setting, inclusion of antiHBc testing for donor screening will definitely remove possible HBV infected donations. Thus though a large number of donations will be rejected, but rejection of these donations will be valuable in reducing the risk of HBV transmission with its potential consequences, particularly among immunocompromised recipients.

Phylogenetic analysis revealed that although HBV/D with subgenotype HBV/D2, HBV/D3 was predominant among OBI cases in this population, HBV/Cs, HBV/Aa and HBV/D1 were also present; this is in accordance with previous reports from other parts of India [20,21,34]. Interestingly, similar to eastern and northern India HBV/D3 was predominant among OBI cases while this subgenotype was mainly found among drug addicts of Europe [35]. The most common amino acid substitution found within MHL region was T125 M (44/60), T118V (11/60) and A128V (11/60) found in HBV/D2 and HBV/D3. A stop codon was found in HBV/D2 at position 69^th ^amino acid which results in a truncated HBsAg gene lacking the total 'a' determinant region, which might be a reason for HBsAg negativity making the surface gene nonfunctional. This mutation was previously documented in subgenotype D1 [36]. Only one sample had M133T substitution in genotype HBV/D, previously described to be associated with diagnostic and vaccine failure [37]. None of the other substitutions found were known to interfere in HBsAg assay. Consequently, a low HBV viral load seems the most likely explanation for our HBsAg negative/DNA positive samples. Some of these had substitutions that also caused amino acid mutations in the overlapping reverse transcriptase (RT) domain in the pol gene; therefore, the typically low level of HBV DNA observed in occult HBV infections, may be related to the mutation in RT domain which in turn resulted in low level of protein synthesis, as reported from eastern India [38].

In conclusion, our study underscores the high rate of exposure of HBV to the blood donors of Behrampur, Ganjam, a high HIV prevalent district of India. One in every 11 blood donors who are HBsAg negative/antiHBc-positive and one in every 3 antiHBc positive/antiHBs positive donors, have OBI, with possibility of transmission of HBV to transfusion recipients. Our results suggest inclusion of antiHBc testing for donor screening. Although addition of antiHBc testing, will lead to rejection of a large number of donor units; it will definitely eliminate HBV infected donations. This will help in reducing transfusion associated transmission due to OBI in this resource poor setting with its potential consequences, especially among the immunocompromised population. The significance of high rate of OBI should be monitored by further studies to evaluate the transmissibility and infectivity of OBI by blood transfusion. These data might lead to improvements in donor screening in the blood banks of India.

## Competing interests

The authors declare that they have no competing interests.

## Authors' contributions

RP designed and performed the majority of experiments; AB, SD, AB and PKC were involved in experiment design; BP coordinated and provided the collection of human materials and editing the manuscript; PM, SC and RC participated in designing the study, preparation and editing the manuscript. All authors have read and approved the final manuscript.
